# Prevalence of Proteinuria and Albuminuria in an Obese Population and Associated Risk Factors

**DOI:** 10.3389/fmed.2018.00122

**Published:** 2018-04-30

**Authors:** Jordan L. Rosenstock, Max Pommier, Guillaume Stoffels, Satyam Patel, Michael F. Michelis

**Affiliations:** ^1^Division of Nephrology, Lenox Hill Hospital, Northwell Health, New York, NY, United States; ^2^Division of Biostatistics, Lenox Hill Hospital, Northwell Health, New York, NY, United States

**Keywords:** obesity, proteinuria prevalence, albuminuria prevalence, risk factors proteinuria, diabetes complications, hypertension

## Abstract

Obesity has been increasingly recognized as a risk factor for kidney disease and both proteinuria and microalbuminuria have been associated with obesity. The actual prevalence of microalbuminuria and proteinuria in obese patients in the United States (US) has not been clearly described in the literature. Furthermore, obesity is associated with risk factors of kidney disease, such as diabetes and hypertension (HTN), and the prevalence of proteinuria and albuminuria excluding these risk factors is uncertain. In this study, we collected urine albumin/creatinine and urine protein/creatinine ratios on obese patients undergoing bariatric surgery to determine the prevalence of albuminuria and proteinuria in obese patients with and without associated diabetes and HTN. The study included 218 obese patients undergoing bariatric surgery at a New York City hospital. The mean age was 42.1 ± 11.3 years. The mean body mass index (BMI) was 43.9 ± 8.1. Diabetes (DM) was present in 25%. HTN was present in 47%. The prevalence of proteinuria and albuminuria was 21% (95% CI: 15.8–27.1%) and 19.7% (95% CI: 14.2–26.2%) respectively. Among those without DM but who had HTN, 22.6% (95% CI: 12.9–35) had proteinuria and 17% (95% CI 8.4–30.9) had albuminuria. Of patients with neither DM nor HTN, 13.3% (95% CI: 7.3–21.6) and 11% (95% CI: 5–17%) had proteinuria and albuminuria, respectively. Diabetics had a significantly higher prevalence of proteinuria and albuminuria than the non-diabetic groups. The non-diabetic groups did not differ significantly from each other in terms of prevalence of proteinuria and albuminuria. The BMI for diabetics did not differ from non-diabetics. On multivariate analysis, only the presence of diabetes was associated with proteinuria and albuminuria. BMI, age, and HTN were not predictive. In conclusion, we found a relatively high prevalence of microalbuminuria and proteinuria in an urban, US, obese population undergoing bariatric surgery. When diabetics were excluded, there was a lower prevalence. Even patients who had neither diabetes nor HTN, still, however, had much greater amounts than seen in the general US population, likely reflecting an adverse effect of obesity itself on renal physiology.

## Introduction

Obesity has been increasingly recognized as a risk factor for kidney disease (1, 2). Increased body mass index (BMI) has been linked to glomerular hyperfiltration (3). Both proteinuria and microalbuminuria have been associated with obesity (2). Pathologic changes in the kidney include the development of focal segmental glomerulosclerosis and glomerulomegaly (4). Proteinuria has been shown to improve with a weight loss in a number of series (1, 5). Increased BMI has been linked to a loss of renal function as well as increased risk of end stage renal disease (1, 2). Despite all of this evidence, the actual prevalence of microalbuminuria and proteinuria in obese patients in the United States (US) has not been clearly described in the literature. Furthermore, obesity is associated with risk factors for the development of kidney disease, such as diabetes and hypertension (HTN), and the prevalence of proteinuria and albuminuria without these risk factors is not certain. In this study, we collected urine albumin/creatinine and urine protein/creatinine ratios on obese patients undergoing bariatric surgery to determine the prevalence of albuminuria and proteinuria in an obese population. We also wanted to evaluate how the prevalence changed based on the presence or absence of associated diabetes and HTN, in order to assess the relative importance of these factors in obese patients, in contributing to proteinuria and albuminuria.

## Material and Methods

Consecutive patients undergoing bariatric surgery at a single hospital in New York City were recruited. Urine samples were collected from each patient prior to surgery and sent for urine albumin/creatinine and urine protein/creatinine ratios in order to assess for both overall urine protein excretion as well as more specifically albuminuria. Albuminuria was defined as an albumin to creatinine ratio of more than 30 mg per gram of creatinine and proteinuria as a protein to creatinine ratio of greater than 150 mg per gram of creatinine. Patients with evidence of a urinary tract infection or active urinary sediment were excluded. All patients had BMI calculated preoperatively. Charts were reviewed for associated diagnoses including diabetes and HTN as well as ACE inhibitor or angiotensin receptor blocker (ARB) use.

For analysis of risk factors, patients were divided into three groups: (1) diabetics with and without HTN (DM), (2) hypertensives without diabetes (HTN), and (3) those with neither. There were too few diabetics without HTN to analyze separately. We did not have data on other diagnoses such as glomerulonephritis or lupus that could have contributed to renal disease, but patients with evidence of active urinary sediment were excluded.

### Statistical Analysis

Confidence intervals for proportions were obtained using the Clopper–Pearson method. Continuous variables were compared across the three patient groups using the Kruskal–Wallis or one-way ANOVA test (as appropriate); categorical variables were compared across the three groups using the Chi-square or Fisher’s exact test (as appropriate). *Post hoc* tests were accomplished using the Mann–Whitney or *t*-test for continuous variables and Chi-square test or Fisher’s exact test for categorical variables. In the case of multiple comparisons, the significance level was adjusted using the Bonferroni method. Two separate multivariable logistic regression model were used to assess the relationship between proposed factors and proteinuria or albuminuria. Unless otherwise specified, a result was considered statistically significant at the *p* < 0.05 level of significance. All analyses were performed using SAS version 9.4 (SAS Institute, Cary, NC, USA).

## Results

The study included 218 patients. Two hundred fourteen patients had measurement of urine protein/creatinine and 183 patients had urine albumin/creatinine measured. The mean age was 42.1 ± 11.3 years. The mean BMI was 43.9 ± 8.1. Diabetes was present in 25%. HTN was present in 47% of all patients. The use of ACE inhibitor/ARB was 26.2% overall in our population and was present in 38.5 and 46.2% in patients with proteinuria and albuminuria respectively. Baseline characteristics for all patients and for each patient group are shown in Table 1. Diabetics were slightly older and had more frequent use of ACEI/ARB than the other groups. The BMI for diabetics did not differ from non-diabetics.

**Table 1:** Baseline characteristics.

	Overall (*n* = 218)	DM (*n* = 55)	Hypertension (HTN) only (*n* = 62)	No HTN, no DM (*n* = 101)	*p*-Value
Age, mean ± SD	42.1 ± 11.3	47.1 ± 11.7	45.5 ± 9.8	37.2 ± 10.0	<0.0001
Body mass index, mean ± SD	43.9 ± 8.1	43.4 ± 10.1	43.7 ± 7.7	44.4 ± 7.3	0.21
ACEi/angiotensin receptor blocker (%)	57 (26.2%)	30 (54.6%)	27 (43.4%)	0 (0%)	<0.0001
Male (%)	64 (29.4%)	17 (30.9%)	17 (27.4%)	30 (29.7%)	0.91

The prevalence of proteinuria and albuminuria overall was 21% (95% CI: 15.8–27.1%) and 19.7% (95% CI: 14.2–26.2%), respectively. Among patients with proteinuria, the median amount was 223 mg (IQR: 183–357). Of those with albuminuria, the median urine albumin was 83 mg (IQR: 55–203). Of patients with albuminuria, 80.6% had microalbuminuria alone, and 19.4% had macroalbuminuria. Of the patients with proteinuria, 40% had DM as did 48.6% of the patients with albuminuria.

In analyzing the three patient groups, among DM patients (with or without HTN), 33.3% (95% CI: 21.1–47.5) had proteinuria and 41.5% (95% CI: 26.3–57.9) had albuminuria. Among those without DM but who had HTN, 22.6% (95% CI: 12.9–35) had proteinuria and 17.7% (95% CI 8.4–30.9) had albuminuria. Of patients with neither DM nor HTN, 13.3 (95% CI: 7.3–21.6%) and 11.0% (95% CI: 4.9–18.9%) had proteinuria and albuminuria, respectively. The percentages of proteinuria and albuminuria in each patient group are shown in Table 2. Diabetics had a significantly higher prevalence of albuminuria than non-diabetics with HTN (*p* = 0.012^†^) and non-diabetics with no HTN (*p* < 0.0001^†^). Diabetics had a significantly higher prevalence of proteinuria than non-diabetics with no HTN (*p* = 0.003^†^) but the increased prevalence did not reach statistical significance when compared with non-diabetics with HTN (0.20). The non-diabetic groups did not differ significantly from each other in terms of prevalence of proteinuria and albuminuria.

**Table 2:** Prevalence of proteinuria and albuminuria in each patient group.

	Overall (*n* = 218)	DM (*n* = 55)	Hypertension (HTN), no DM (*n* = 62)	No HTN, no DM (*n* = 101)	*p*-Value
Proteinuria, *n* (%, CI)	45 (21.0%, 15.8–27.1)	18 (33.3%, 21.1–47.5)	14 (22.6%, 12.9–35)	13 (13.3%, 7.2–21.6)	0.01
Albuminuria, *n* (%, CI)	36 (19.7%, 14.2–26.2)	17 (41.5%, 26.3–57.9)	9 (17.7%, 8.4–30.9)	10 (11.0%, 4.9–18.9)	0.0002

DM patients as a group had higher median amounts of proteinuria and albuminuria than the group without diabetes and without HTN. However, on *post hoc* analysis, there was not enough evidence to conclude that they differed from the median amount found in non-diabetics with HTN.

The BMI did not significantly differ between those with and without proteinuria or albuminuria (*p* = 0.74 and *p* = 0.64, respectively). Multivariable analysis showed that, among all included factors (DM, age, HTN, and ACE inhibitor/ARB use), DM was the only significant factor for proteinuria (*p* = 0.0001) and albuminuria (*p* = 0.0001).

## Discussion

Obesity has been increasingly recognized as a risk factor for renal disease but the exact prevalence of albuminuria and proteinuria, especially accounting for associated risk factors, is not clear. Two studies from France were published in the 1990s that did report on prevalence of albuminuria in overweight and obese patients. One study found a prevalence of 7.1% of microalbuminuria and 2.7% of macroalbuminuria in patients without diabetes or HTN (6). The other study, which also did not include diabetic patients but included hypertensives, found no cases of proteinuria but did find a higher incidence of microalbuminuria in those with HTN (19 versus 9.7%) (7). Unlike our study, both of these studies included non-obese, overweight patients whose BMI was greater than 27. The third and by far largest study addressing prevalence was the PREVEND study from the Netherlands, published in 2003 (8). That study, which excluded diabetics, found a prevalence of microalbuminuria of 21 or 13% depending on central or peripheral obesity patterns (with central obesity having a higher incidence). Approximately half of the patients in the study had HTN, but the prevalence of albuminuria with and without HTN was not provided in the obese population studied by PREVEND.

Our study is the first, to our knowledge, in an American urban population. Our study, unlike previous studies, also only included truly obese patients with BMI ≥ 30. Still, the prevalence of albuminuria is remarkably similar to the findings from France more than 20 years ago in an obese and overweight population without diabetes or HTN (6, 7). This is also one of the first studies on an obese population to focus on proteinuria in addition to albuminuria in an obese population.

NHANES 3 data from 2002 showed that in the US population, 5.1% of people without diabetes or HTN had albuminuria and 0.3% had macroalbuminuria (9). Thus, even though it is clear that concomitant diabetes or HTN greatly increases the risk of albuminuria and proteinuria, it is evident from our study that obesity itself more than doubled the prevalence of albuminuria when compared with general population. This is consistent with a recent report that “metabolically healthy obesity” without HTN or evidence of metabolic syndrome is still associated with the development of chronic kidney disease (10).

We found a higher prevalence of proteinuria and albuminuria when compared with previous reports of renal disease in diabetics. For example, UKPDS reported 28% microalbuminuria and 7% proteinuria prevalence after 15 years of type 2 diabetes (11). A report in type 1 diabetes found 34% microalbuminuria and 15% proteinuria after 18 years of median follow up (12). The higher prevalence in our population, especially when compared with type 2 diabetics, could be due to the concomitant obesity. Indeed, a published analysis of the Diabetes Control and Complications Trial found that both waist circumference and BMI were risk factors for the development of incident microalbuminuria over 5.8 years in type 1 diabetics (13). Unfortunately, in our population, we do not have information of percentages on type 1 versus type 2 diabetes, the duration of diabetes, nor the hemoglobin A1Cs of our patients in order to further refine risks factors for diabetic kidney disease.

Approximately, a quarter of our patients were on ACEI or ARB and it is likely that the prevalence of proteinuria and albuminuria as well as the absolute amounts of albumin and protein excretion would be affected by the use of these medicines. Indeed, it is likely that the extent of renal disease in our patients is underestimated by our study. A study from Spain showed that renal pathological changes, including mesangial matrix increase, mesangial proliferation, podocyte, and glomerular hypertrophy, could be found in a high percentage of very obese (BMI ≥ 40) patients undergoing bariatric surgery, despite having no clinical evidence of renal disease (14).

A limitation of our study is that albuminuria and proteinuria were determined from a single morning urine sample. However, studies have shown that morning random urine protein/creatinine samples correlate well with 24-h urine protein and may even be more predictive of renal disease progression than 24-hcollections (15). We also did not have information on uric acid levels, triglycerides in order to assess other components of the metabolic syndrome, which also may add to the risks for kidney disease. We did not measure renin angiotensin aldosterone activation, leptin, or other adipokines which could have a bearing on renal pathophysiology. Finally, we did not assess for obesity pattern, peripheral or central, as was done in the PREVEND study.

Also, it must be noted that our patients were specifically obese patients who were undergoing bariatric surgery. These patients likely differ in important ways from a non bariatric surgery obese population. On the one hand, these patients were cleared for major elective surgery, which presumably excluded patients at highest cardiovascular risk. On the other hand, the fact that they decided to undergo the surgery may reflect a concern for risk factors such as concomitant DM and HTN and other complications of obesity that could impact renal disease.

In conclusion, we found a relatively high prevalence of albuminuria and proteinuria in an urban, US, obese population undergoing bariatric surgery. When diabetics were excluded, there was a lower prevalence and the mean amounts of proteinuria and albuminuria were less. Non-diabetic obese patients with HTN had more albuminuria and proteinuria than those without HTN, but this did not reach statistical significance in our population. Obese patients who had neither diabetes nor HTN, in other words without another apparent cause of renal disease, still had much greater amounts than seen in the general United States population as described in the NHANES data. This likely reflects an adverse effect of obesity itself on renal physiology. Considering that albuminuria and proteinuria are well recognized risk factors for progressive renal disease as well as cardiovascular disease, this highlights one reason among many for the crucial need to reverse the rising worldwide obesity epidemic.

## Ethics Statement

All procedures performed in studies involving human participants were in accordance with the ethical standards of the institutional and/or national research committee and with the 1964 Helsinki declaration and its later amendments or comparable ethical standards. The local institutional review board of Northwell health approved the protocol and found it compliant with ethical standards including the requirement for informed consent (IRB study approval #HS15-0574).

## Author Contributions

JR: primary investigator and corresponding author. MP: renal fellow, primary data collector, and assisted manuscript preparation. GS: statistician. SP: renal fellow and assisted with manuscript preparation. MFM: division chair, assisted in guiding project and edited manuscript.

## Conflict of Interest Statement

The authors declare that the research was conducted in the absence of any commercial or financial relationships that could be construed as a potential conflict of interest.
